# Development and cross‐cultural validation of the Japanese version of the Adjustment Disorder‐New Module‐20

**DOI:** 10.1002/pcn5.70104

**Published:** 2025-05-01

**Authors:** Atsuo Nakagawa, Dai Mitsuda, Yusuke Umegaki, Rahel Bachem, Waka Nogami, Ryotaro Higuchi, Aoi Ito, Hiroki Kocha

**Affiliations:** ^1^ Department of Neuropsychiatry St. Marianna University School of Medicine Kawasaki Japan; ^2^ Faculty of Human Life and Environment Nara Women's University Nara Japan; ^3^ Department of Psychology, Psychology and Clinical Intervention University of Zurich Zurich Switzerland

**Keywords:** adjustment disorder, Adjustment Disorder‐New Module‐20 (ADNM‐20), dimensional approach, ICD‐11, psychometric validation

## Abstract

**Aim:**

Adjustment disorder (AjD) is a prevalent mental disorder with evolving definitions in the *International Classification of Diseases*, 11th Revision (ICD‐11). The Adjustment Disorder‐New Module‐20 (ADNM‐20) assesses six AjD symptom clusters based on the ICD‐11 criteria and has demonstrated strong psychometric validity. This study aimed to develop and validate a Japanese version of the ADNM‐20 in the general population.

**Methods:**

A forward–backward translation approach was used to develop the Japanese version of the ADNM‐20. Psychometric properties were assessed through a web‐based survey of 1314 participants, examining factorial validity, construct validity, and internal consistency.

**Results:**

Confirmatory factor analysis supported the theoretical six‐factor model (comparative fit index = 0.923, root‐mean‐square error of approximation = 0.079), with strong correlations between the ADNM‐20 subscales and external measures (e.g., depression PHQ‐9, *r* = 0.71; anxiety GAD‐7, *r* = 0.72). The total ADNM‐20 showed excellent internal consistency (Cronbach's *α* = 0.953).

**Conclusion:**

The Japanese ADNM‐20 is a valid and reliable scale for assessing AjD symptoms in the Japanese general population. Further studies involving clinical populations are warranted to confirm its applicability in clinical settings.

## Graphical abstract

The Japanese version of the Adjustment Disorder‐New Module‐20 (ADNM‐20) is a valid and reliable instrument for assessing adjustment disorder (AjD) symptoms in the Japanese general population.

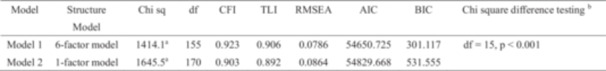

## INTRODUCTION

Adjustment disorder (AjD) is described as a maladaptive response to an identifiable psychosocial stressor or life change, resulting in clinically significant distress or impairment.^1^ AjD is one of the most commonly diagnosed mental disorders.^2^, ^3^ However, the definitions of “AjD” in the *Diagnostic and Statistical Manual of Mental Disorders*, 5th revision (DSM‐5) and the *International Classification of Diseases*, 10th revision (ICD‐10) lack explicit symptom criteria,^4^ often leading to its diagnosis by exclusion. For this reason, AjD has frequently been referred to as the “wastebasket” of the mental disorder diagnostic classifications.^5^, ^6^

The recent revision of the *International Classification of Diseases*, 11th revision (ICD‐11), provides a significant opportunity to refine AjD as a distinct diagnostic category.^7^ The ICD‐11 defines “AjD” by two core symptom clusters that enhance its clinical utility and diagnostic clarity: (1) preoccupation with the stressor (PRE), characterized by recurrent, distressing thoughts about the stressor and its implication; and (2) failure to adapt (FTA), characterized by symptoms such as difficulty concentrating, sleep disturbance, and an inability to recover and find emotional equilibrium.^8^

To improve the clinical assessment of the ICD‐11‐defined AjD, the Adjustment Disorder‐New Module (ADNM) was developed.^9^ This self‐report measure evaluates six symptom clusters: the two core symptom clusters (PRE and FTA) and four accessory clusters (avoidance [AVO], depression [DEP], anxiety [ANX], and impulsivity [IMP]). The ADNM is available in several versions, including the original 29‐item version,^10^ the 20‐item short version (ADNM‐20),^11^ the eight‐item brief version (ADNM‐8),^12^ and the ultra‐brief four‐item version (ADNM‐4).^13^ The ADNM‐20 has been translated into several languages, including German,^14^ Lithuanian,^15^ Chinese,^16^ and French.^17^

While AjD has been studied extensively in Western populations over the past two decades, research on AjD in Japan remains limited. Unique Japanese cultural factors, such as enduring hardship and prioritizing conformity to group norms, may influence the development and manifestation of AjD symptoms.^18^, ^19^ Additionally, Japan's recent economic downturn and increasing global competition have shifted traditional corporate culture toward a more individualistic performance‐based system, while still emphasizing collectivism. This conflicting value system creates unique challenges for Japanese workers as they adjust between these competing demands.^20^ Most prior studies on AjD in Japan have utilized diagnostic classifications based on the ICD‐10^21^, ^22^ or DSM‐5,^23^ highlighting the need for a validated instrument that aligns with the ICD‐11's redefinition of AjD.

This study aimed to adapt and validate the ADNM‐20 in a Japanese population, addressing cross‐cultural differences and contributing to a broader global understanding of AjD.

## METHODS

### Development of the Japanese ADNM‐20

The Japanese version of the ADNM‐20 was developed following the International Society for Pharmacoeconomics and Outcomes Research (ISPOR) guidelines.^24^ The forward translation was initially performed by a bilingual Japanese psychiatrist (A. N.) proficient in the subject matter. Professional native‐English translators experienced in mental health, unaware of the study's objectives, conducted back‐translations. A study psychologist (D. M.) independently reviewed the forward and back translations for semantic equivalence. The original authors of the ADNM‐20 (Professor Andreas Maercker and Rahel Bachem) reviewed the back‐translations for potential biases and discrepancies. This iterative process was repeated twice until the Japanese version achieved semantic equivalence with the original.

### Participants and procedures

Participants were recruited via an online panel designed to represent the Japanese adult population, with stratified random sampling ensuring alignment with age groups and sex based on the 2020 Japanese national census. Eligible participants were adults aged 18 years or older who had experienced at least one stressful life event in the past 2 years that had caused a significant burden within the last 6 months. Participants provided informed consent through an opt‐in procedure and were compensated for their time.

The online survey, conducted in December 2023, took ∼15 min to complete and included demographic data, the Japanese ADNM‐20, and validated measures of depression (Patient Health Questionnaire [PHQ‐9]^25^, ^26^), anxiety (General Anxiety Disorder 7‐item [GAD‐7]^27^, ^28^), and psychological well‐being (World Health Organization Five Well‐Being Index [WHO‐5]^29^, ^30^).

A total of 1314 participants completed the survey, with a mean age of 43.68 years (standard deviation [SD] = 13.32; range = 18–65), and 53.9% were female (*n* = 708). Ethical approval for the study was obtained from the Institutional Review Board of St. Marianna School of Medicine (Approval No. 6288).

### Measures

#### AjD severity

The ADNM‐20 consists of two parts. In the first part, participants reported all stressful life events from the past 2 years that had caused a significant burden during the last 6 months. The list includes seven acute stressors (e.g., death of a loved one, divorce) and nine chronic stressors (e.g., financial problems, conflicts in working life). Participants could also report additional stressors in open‐ended responses. The most distressing stressor identified by the participant was used for further assessment.

In the second part, participants rated 19 items reflecting AjD symptoms and one item assessing functional impairment based on the ICD‐11 criteria, using a four‐point Likert scale (1 = *never* to 4 = *often*). Higher scores indicate greater AjD severity. The original ADNM‐20 has previously demonstrated good psychometric properties in previous research, with Cronbach's *α* of 0.94 for the total score and 0.80–0.90 for the subscales.^31^

#### Depression

Depressive symptoms were assessed using the PHQ‐9, a validated self‐reported measure.^25^ Participants rated the frequency of symptoms over the past 2 weeks on a four‐point Likert scale (0 = *not at all* to 3 = *nearly every day*). Total scores ranged from 0 to 27, with higher scores indicating greater severity. The Japanese version of the PHQ‐9 has demonstrated excellent reliability (Cronbach's *α* = 0.94).^26^

#### Anxiety

Anxiety symptoms were measured using the GAD‐7.^27^ Participants rated symptoms frequency over the past 2 weeks using a four‐point Likert scale (0 = *not at all* to 3 = *nearly every day*). Total scores ranged from 0 to 21, with higher scores indicating greater anxiety severity. The Japanese version of the GAD‐7 has demonstrated good psychometric properties, with Cronbach's *α* as 0.92.^28^

#### Psychological well‐being

The WHO‐5 was used to assess positive mental health.^29^ This validated instrument consists of five items that inquire about the frequency of positive experiences over the past 2 weeks. Responses are recorded on a six‐point Likert scale (0 = *never* to 5 = *always*). Total scores ranged from 0 to 25, with higher scores reflecting better psychological well‐being. The WHO‐5 has shown high reliability in previous studies (Cronbach's *α* = 0.87).^30^

### Statistical analysis

Descriptive statistics characterized the sample and summarized mean scores on the ADNM‐20 and related measures. Demographic and stress‐related characteristics were compared across different levels of AjD symptom severity. Latent class analysis (LCA) classified the sample into three symptom groups—low, moderate, and high—based on response patterns to the ADNM‐20, as reported by Glaesmer et al.^11^ Confirmatory factor analysis (CFA) was conducted to evaluate the structural validity of the Japanese ADNM‐20, testing the six‐factor model proposed by Einsle et al.^10^ and the one‐factor model proposed by Glaesmer et al.^11^ Model fit was assessed using the comparative fit index (CFI), Tucker–Lewis index (TLI), and root‐mean‐square error of approximation (RMSEA). Fit indices were interpreted as follows: RMSEA values below 0.06 indicate a good fit, values between 0.06 and 0.08 indicate a fair fit, values between 0.08 and 0.10 indicate an acceptable fit, and values above 0.10 indicate a poor fit. For CFI and TLI, values above 0.95 indicate a good fit, while values between 0.90 and 0.95 indicate an acceptable fit.^32^ Differences in model fit were evaluated using the *χ* ^2^ different test and information criteria comparison (Akaike information criterion [AIC] and the Bayesian information criterion [BIC]). A lower value of AIC and BIC indicate a better fit.

Pearson's correlation coefficients (*r*) were used to examine correlations between the subscale scores of the six ADNM‐20 clusters (PRE, FTA, AVO, DEP, ANX, and IMP). Convergent validity was evaluated by calculating correlations between the ADNM‐20 and the PHQ‐9 (depression) and GAD‐7 (anxiety) to assess the level of similarity of the construct. Divergent validity was assessed by calculating correlations between the ADNM‐20 and the WHO‐5 (psychological well‐being), a distinct construct to the ADNM‐20. Correlation coefficients were interpreted as follows: values above 0.8 were considered very strong, values between 0.6 and 0.8 were considered strong, values between 0.4 and 0.6 were considered moderate, values between 0.2 and 0.4 were considered weak, while values below 0.2 were considered very weak.^33^

Internal consistency was assessed using Cronbach's *α* for the total scale and subscales. Cronbach's *α* was interpreted as follows: values above 0.9 were considered excellent, values between 0.8 and 0.9 were considered good, and values between 0.7 and 0.8 were considered acceptable.^34^ All statistical analyses were conducted using JMP Pro Version 17 (SAS Institute Inc.).

## RESULTS

### Descriptive analysis

Table 1 presents the descriptive statistics for the 1314 participants, along with an overview of demographic and stress‐related characteristics across the three groups identified through LCA. The mean total score on the ADNM‐20 for the entire sample was 55.6 (SD = 13.9). Based on the proposed ADNM‐20 cut‐off score of 47.5,^31^ 73.6% (*n* = 967) of participants were classified as at risk of AjD. The most common occupation was company employee, accounting for 43.7% (*n* = 574) of the entire sample. Regarding life stressors, financial difficulties were the most frequently reported (39.8%, *n* = 523), followed by work–life conflicts (33.4%, *n* = 439), and familial conflicts (28.4%, *n* = 373).

**Table 1:** Demographic and stress‐related characteristics across AjD symptom severity levels.^a^

Variables	Total		Low symptom		Moderate symptom		High symptom		Statistical test^b^	
	*n* = 1314		*n* = 246 (18.72%)		*n* = 647 (49.24%)		*n* = 421 (32.04%)			
	Mean	SD	Mean	SD	Mean	SD	Mean	SD	*F*	*p*
Age (years)	43.68	13.32	45.98	13.75	44.18	13.20	41.57	12.97	9.52	<0.0001
ADNM‐20										
Total score	55.62	13.90	35.23	0.44	53.93	0.27	70.13	7.15	2068.60	<0.0001
Core symptoms										
Preoccupation with the stressor	11.21	2.93	7.19	0.11	10.85	0.07	14.11	0.08	1356.33	<0.0001
Failure to Adapt	9.79	3.09	5.68	0.12	9.44	0.07	12.73	0.09	1105.94	<0.0001
Accessory symptoms										
Avoidance	10.58	2.85	7.04	0.13	10.39	0.08	12.94	0.10	684.78	<0.0001
Depressed mood	8.08	2.25	5.24	1.66	7.79	1.38	10.18	1.42	924.69	<0.0001
Anxiety	5.48	1.59	3.52	1.21	5.30	0.99	6.91	1.08	804.94	<0.0001
Impulse disturbance	7.76	2.38	4.74	1.67	7.51	1.40	9.90	1.75	849.83	<0.0001
PHQ‐9 score	8.50	6.73	2.73	0.33	6.89	0.20	14.35	6.54	451.94	<0.0001
GAD‐7 score	6.39	5.51	1.63	0.26	4.92	0.16	11.42	0.20	521.05	<0.0001
WHO‐5 score	10.96	5.71	14.71	5.10	11.41	5.05	8.09	0.25	129.19	<0.0001

	***N***	**%**	***N***	**%**	***N***	**%**	***N***	**%**	*χ* ^2^	***p***
Gender (Female)	708	53.9	150	21.19	333	47.03	225	31.78	6.53	0.038
Current occupation										
Public employee	59	4.49	11	18.64	35	59.32	13	22.03	3.21	0.201
Company executive	8	0.61	2	25.00	2	25.00	4	50.00	1.94	0.379
Company employee	574	43.68	100	17.42	298	51.92	176	30.66	3.01	0.222
Home business owner	38	2.89	10	26.32	20	52.63	8	21.05	2.77	0.250
Freelancer	28	2.13	7	25.00	13	46.43	8	28.57	0.76	0.685
Homemaker	160	12.18	38	23.75	71	44.38	51	31.88	3.34	0.189
Part‐time employee	241	18.34	47	19.50	115	47.72	79	32.78	0.29	0.867
Student	58	4.41	9	15.52	26	44.83	23	39.66	1.67	0.433
Other occupation	40	3.04	4	10.00	23	57.50	13	32.50	2.25	0.325
Unemployed	108	8.22	18	16.67	44	40.74	46	42.59	5.84	0.054
Life stressors										
Acute stressors										
Death of a loved one	112	8.52	39	34.82	50	44.64	23	20.54	22.53	<0.0001
Termination of important leisure activity	32	2.44	7	21.88	16	50.00	9	28.13	0.34	0.846
Moving to a new home	34	2.59	9	26.47	18	52.94	7	20.59	2.65	0.266
Adjustment due to retirement	25	1.90	4	16.00	14	56.00	7	28.00	0.47	0.792
Divorce/separation	14	1.07	1	7.14	6	42.86	7	50.00	2.56	0.278
Serious accident	11	0.84	1	9.09	6	54.55	4	36.36	0.68	0.713
Assault	2	0.15	0	0.00	1	50.00	1	50.00	0.58	0.749
Chronic stressors										
Financial problems	279	21.23	52	18.64	136	48.75	91	32.62	0.06	0.973
Conflict in work life	216	16.44	27	12.50	114	52.78	75	34.72	6.58	0.037
Familial conflict	198	15.07	23	11.62	100	50.51	75	37.88	8.84	0.012
Pressure to meet deadlines/time pressure	79	6.01	15	18.99	43	54.43	21	26.58	1.25	0.537
Too much/too little work	84	6.39	15	17.86	43	51.19	26	30.95	0.14	0.933
Illness of a loved one	70	5.33	11	15.71	41	58.57	18	25.71	2.59	0.274
Own serious illness	67	5.10	22	32.84	23	34.33	22	32.62	10.71	0.005
Conflict with the neighbors	24	1.83	5	20.83	11	45.83	8	33.33	0.13	0.938
Unemployment	15	1.14	3	20.00	4	26.67	8	53.33	3.73	0.155
Other stressful events	52	3.96	12	23.08	21	40.38	19	36.54	1.75	0.416

Abbreviations: ADNM‐20, Adjustment Disorder‐New Module‐20; AjD, adjustment disorder; GAD‐7, Generalized Anxiety Disorder Seven‐Item (measures anxiety); PHQ‐9, nine‐item Patient Health Questionnaire (measures depression); SD, standard deviation; WHO‐5, World Health Organization‐Five Well‐Being Scale (measures psychological well‐being.

^a^ Demographic and stress‐related characteristics were compared across different levels of AjD symptom severity. Latent class analysis classified the sample into three symptom groups—low, moderate, and high—based on response patterns to the ADNM‐20.

^b^ Continuous data were analyzed using one‐way analysis of variance, while categorical data were examined using *χ* ^2^ tests.

Among the total sample, 246 participants (18.7%) were classified into the low‐symptom group, with a mean ADNM‐20 score of 35.2, 647 participants (49.2%) into the moderate‐symptom group (mean ADNM‐20 score of 53.9), and 421 participants (32.0%) were classified into the high‐symptom group (mean ADNM‐20 score of 70.1). Across the three subgroups, the mean PHQ‐9 and GAD‐7 scores were highest in the high‐symptom group, whereas the mean WHO‐5 scores were highest in the low‐symptom group. Significant differences were observed in gender and age distribution across the subgroups, with female participants more prevalent in the moderate‐symptom group and younger participants more prevalent in the high‐symptom group. Regarding the frequency of acute stressors, there were no significant differences in the distribution except for “death of a loved one,” which was more frequently reported in the moderate‐symptom group. As for chronic stressors, “conflict in work life,” “familial conflict,” and “own serious illness” were frequently reported in the moderate‐symptom group.

### Factorial validity

The results of the CFA are shown in Table 2. Both the six‐factor model (Model 1) and the one‐factor model (Model 2) demonstrated acceptable fit indices, with CFI and TLI values of approximately 0.91. Model 1 exhibited a fair fit, with an RMSEA below 0.08, while Model 2 showed an acceptable fit. Although *χ* ^2^ tests were statistically significant for both models, it is important to note that large sample sizes can increase the likelihood of statistically significant findings, even for minor model misspecifications.^35^ However, further model comparisons provided additional support for the six‐factor structure. The *χ* ^2^ difference test (*p* < 0.001) and the information criteria (lower AIC and BIC values for Model 1) both indicated that the six‐factor model (Model 1) provided a superior fit than that of the one‐factor model (Model 2).

**Table 2:** Goodness‐of‐fit indices for structural models (*n* = 1314).

Model	Structure model	*χ* ^2^	df	CFI	TLI	RMSEA	AIC	BIC	*χ* ^2^ difference testing^a^
Model 1	6‐factor model	1414.1*	155	0.923	0.906	0.0786	54650.725	301.117	*df* = 15, *p* < 0.001
Model 2	1‐factor model	1645.5^a^	170	0.903	0.892	0.0864	54829.668	531.555	

Abbreviations: AIC, Akaike information criterion; BIC, Bayesian information criterion; CFI, comparative fit index; df, degrees of freedom; RMSEA, root‐mean‐square error of approximation; TLI, Tucker–Lewis index.

^a^ *χ* ^2^ difference between the six‐factor model and the one‐factor model, a significant value implies a significantly worse fit for the one‐factor model compared to the six‐factor model.

* *p* < 0.001.

### Construct validity

As shown in Table 3, the six ADNM‐20 subscales exhibited strong correlations with the total score, ranging from 0.66 to 0.91. Furthermore, strong correlations between the ADNM‐20 and the PHQ‐9 (*r* = 0.71) and GAD‐7 (*r* = 0.72) provided evidence of convergent validity, demonstrating alignment with related constructs. Conversely, the moderate negative correlation between the ADNM‐20 and the WHO‐5 (*r* = −0.47) supported divergent validity, confirming that the ADNM‐20 measures a construct distinct from positive psychological well‐being. When the ADNM‐20 total score was substituted with the DEP and ANX subscales, significant correlations were still observed with depression (PHQ‐9: *r* = 0.68) and anxiety (GAD‐7: *r* = 0.64), respectively. However, these subscale correlations were weaker than those obtained using the total ADNM‐20 score.

**Table 3:** Pearson's correlation (*r*) between the six ADNM‐20 subscales and the total scale (*n* = 1314).

	Failure to Adapt	Preoccupation	Avoidance	Depression	Anxiety	Impulsivity
Failure to Adapt	1.00					
Preoccupation	0.76	1.00				
Avoidance	0.69	0.72	1.00			
Depression	0.77	0.77	0.66	1.00		
Anxiety	0.73	0.77	0.72	0.72	1.00	
Impulsivity	0.74	0.76	0.66	0.69	0.68	1.00
Total scale	0.90	0.91	0.86	0.87	0.86	0.86

*Note*: All *p*‐values of Pearson's correlations were significant (*p* < 0.0001).

Abbreviation: ADNM‐20, Adjustment Disorder‐New Module‐20.

### Internal consistency

The total ADNM‐20 scale demonstrated excellent internal consistency, with Cronbach's *α* = 0.953. Subscale reliability ranged from acceptable to good: PRE (Cronbach's *α* = 0.856), FTA (Cronbach's *α* = 0.837), AVO (Cronbach's *α* = 0.786), DEP (Cronbach's *α* = 0.758), ANX (Cronbach's *α* = 0.693), and IMP (Cronbach's *α* = 0.809).

## DISCUSSION

The present study aimed to develop and validate a Japanese version of the ADNM‐20, a self‐report measure designed to assess AjD symptoms based on the ICD‐11 criteria. The findings demonstrated that the Japanese version of the ADNM‐20 exhibits good psychometric properties, including factorial validity, construct validity, and internal consistency.

The CFA supported the six‐factor structure of the ADNM‐20, consistent with the original conceptualization of the instrument. This result confirms that the Japanese version effectively measures the six distinct symptom clusters of AjD as defined by the ICD‐11. These findings align with previous CFA‐based validation studies by Einsle et al.^10^ and others^16^, ^17^, ^36^ that support the six‐factor structure of the ADNM‐20 and further validate the cross‐cultural applicability of the ADNM‐20. Notably, the original development of the ADNM by Einsle et al.^10^ employed principal component analysis to explore its structure, offering an empirical foundation for subsequent theory‐driven CFA approaches. While our results favored the six‐factor model, a recent study by Quero et al.^37^ supports a hierarchical model in which the six symptom dimensions load onto a single higher‐order construct of AjD severity, indicating that the dimensions are distinct yet highly interrelated. Future research should consider applying exploratory methods, such as exploratory factor analysis, in Japanese samples to further assess cross‐cultural applicability and evaluate the parsimony of alternative models.

The excellent internal consistency of the total scale (Cronbach's *α* = 0.953) and the good‐to‐acceptable internal consistency of the subscales (ranging from 0.693 to 0.856) further support the instrument's reliability. These results align with studies on other language versions of the ADNM‐20, further underscoring the measure's consistency across cultural adaptations.^14^, ^15^, ^16^, ^17^, ^36^ However, while a high Cronbach's *α* for the total scale (Cronbach's *α* = 0.953) indicates excellent reliability, values exceeding 0.9 may also indicate potential item redundancy.^34^ To address this concern, we examined inter‐item and item‐total correlations and found that none of the subscale *α* scores exceeded 0.9, suggesting that redundancy is less likely within the subscales.

The construct validity of the Japanese ADNM‐20 was also strong. Strong correlations between the subscale scores of the six symptom clusters and the total score (ranging from 0.66 to 0.91) suggest that the subscales effectively measure distinct yet related dimensions of AjD. Furthermore, significant correlations with established measures of depression (PHQ‐9; *r* = 0.71) and anxiety (GAD‐7; *r* = 0.72) provide evidence of concurrent validity, indicating that the ADNM‐20 captures constructs closely related to AjD. The moderate negative correlation with the WHO‐5 (*r* = −0.47) supports divergent validity, confirming that the ADNM‐20 assesses a construct distinct from positive well‐being. One possible explanation for the stronger correlations between the total ADNM‐20 score and the PHQ‐9/GAD‐7 scores, compared to the DEP and ANX subscales, is the broader symptom coverage of the total scale (ADNM‐20 total scale vs. DEP subscale: PHQ‐9, *r* = 0.71 vs. *r* = 0.68; ADNM‐20 total scale vs. ANX subscale: GAD‐7, *r* = 0.72 vs. *r* = 0.64). While the DEP and ANX subscales primarily assess depressive and anxious mood, the total ADNM‐20 score encompasses a broader range of AjD symptoms, including restlessness (Item 8), difficulty concentrating (Item 10), and sleep disturbances (Item 13). These symptoms are commonly observed in individuals with maladaptive stress responses and are also assessed in the GAD‐9 and PHQ‐9, which may partly explain the stronger associations between the total ADNM‐20 score and measures of depression and anxiety.

Demographic and stress‐related characteristics were compared across different levels of AjD symptom severity. Mean ADNM‐20, PHQ‐9, and GAD‐7 scores were highest in the high‐symptom group and lowest in the low‐symptom group across the three latent classes. These findings align with previous studies,^11^ suggesting that the ADNM‐20 score reflects a continuum of AjD symptom intensity. We also observed that female participants were more prevalent in the moderate‐symptom group, consistent with previous findings from the German general population.^11^ One explanation for the gender differences in depression and anxiety is that females tend to ruminate more frequently than males, becoming preoccupied with repetitive negative emotions and problems rather than engaging in active problem‐solving.^38^ Additionally, we found that younger participants were more prevalent in the high‐symptom group. The relationship between age and mental disorder risk remains a topic of debate, warranting further research to clarify this association. While acute stressors demonstrated a negligible association with the distribution of AjD symptoms, several chronic stressors, such as work–life conflict, family conflict, or own serious illness, were associated with moderate AjD symptoms. These findings are consistent with previous research utilizing the same assessment instrument, though with variations in the analytical approaches.^11^, ^14^

This study has several notable strengths. First, the large, representative sample of the Japanese population facilitated robust psychometric analyses, strengthening the reliability and generalizability of the findings. Second, the use of well‐established, validated instruments for comparison, such as the PHQ‐9, GAD‐7, and WHO‐5, enhanced the credibility of the construct validity analysis. Additionally, the rigorous forward–backward translation process ensured the semantic equivalence of the Japanese version with the original ADNM‐20.

However, several limitations should be acknowledged. Using an online panel for participant recruitment may limit the generalizability of the results to the broader Japanese population, as online samples may differ in demographic or psychological characteristics from the general population. Additionally, while the translation process was rigorous, subtle cultural nuances may still influence how certain items are interpreted. The study also did not include a clinical population or employ structured interviews based on the ICD‐11 criteria, which limits the ability to assess criterion validity. Future research is needed to evaluate the validity of the Japanese ADNM‐20 as a screening tool by incorporating semi‐structured interviews based on the ICD‐11 AjD criteria in clinical populations to enable a more comprehensive assessment. Moreover, additional exploratory approaches, such as exploratory factor analysis, should be applied to further assess the cross‐cultural robustness and structural parsimony of the scale.

## CONCLUSION

The Japanese version of the ADNM‐20 is a valid and reliable instrument for assessing AjD symptoms in the Japanese general population. It demonstrated good psychometric properties, including factorial validity, construct validity, and internal consistency. These findings support its utility as a valuable tool for evaluating an individual's AjD symptomatology. Future research should examine its clinical utility, particularly in clinical populations, to further establish its applicability in diagnostic and therapeutic contexts.

## AUTHOR CONTRIBUTION

**Atsuo Nakagawa**: Conceptualization; study design; questionnaire development; survey conduction; data curation; data analysis; drafting the initial manuscript; reviewing and editing the manuscript; funding acquisition. **Dai Mitsuda, Waka Nogami, Ryotaro Higuchi, Aoi Ito,** and **Rahel Bachem**: Questionnaire review; reviewing and editing the manuscript. **Yusuke Umegaki**: Data analysis; reviewing and editing the manuscript. **Hiroki Kocha:** Supervision; reviewing and editing the manuscript. All authors contributed to and have approved the final version of the manuscript.

## CONFLICT OF INTEREST STATEMENT

The authors declare no conflict of interest.

## ETHICS APPROVAL STATEMENT

This study was conducted in compliance with the provisions of the Declaration of Helsinki, and the study protocol was approved by the Institutional Review Board of St. Marianna University School of Medicine (approval no: 6288).

## PARTICIPANT CONSENT STATEMENT

Informed consent was obtained from all study participants via a web‐based platform.

## CLINICAL TRIAL REGISTRATION

N/A.

## Supporting information

Supporting information.

## Data Availability

The data that support the findings of this study are available from the corresponding author upon reasonable request.
