# Supplementary material for: Development and cross‐cultural validation of the Japanese version of the Adjustment Disorder‐New Module‐20
Source: PCN Rep. 2025 May 1;4(2):e70104. doi: 10.1002/pcn5.70104 (PMC12045789; doi:10.1002/pcn5.70104)
Supplement: Supplementary file 1 — Supporting information. [file PCN5-4-e70104-s001.pdf]

ADNM-20 質問票

Adjustment Disorder – New Module 20

Q1. 以下に、様々な生活上のストレスフルな出来事(状況変化)が示されています。この2年の間に起きた出来事（状況変化）の中で、現在あなたにとって非常に強い負担となっているもの、またはこの6ヶ月、あなたに非常に強い負担をかけていたものに〇をつけてください。
該当する出来事（状況変化）は、当てはまるものはいくつ〇をつけても構いません。

ストレスフルな出来事（状況変化）	該当する（〇）
01. 離婚／別居	
02. 家族との衝突や葛藤	
03. 仕事での衝突や葛藤	
04. 近隣住民との衝突	
05. 大切な人の病気	
06. 大切な人の死	
07. 退職に伴う生活変化への適応	
08. 失業	
09. 過小な業務量／過大な業務量	
10. 期限を守らなければならないプレッシャー／時間的プレッシャー	
11. 新しい家への引っ越し・転居	
12. 経済的な問題	
13. 自身の重大な病気	
14. 重大な事故	
15. 暴行	
16. 大切な余暇活動の終了	
17. その他ストレスの多い出来事（ご記入ください: ）	
18. その他ストレスの多い出来事（ご記入ください: ）	

上の欄で〇をつけた出来事（状況変化）は、私たちのウェルビーイング・健康や行動に大きな影響をもたらします。最も強い負担となった出来事（状況変化）の番号を下の欄に記入ください。

最も強い負担となった出来事（状況変化）	

Q2. Q1で確認した最も強い負担となった出来事（状況変化）によって引き起こしうる反応についてのさまざまな記述が以下に示されています。

1）各項目があなたにどのくらい頻繁に起こるかについて、最も近いものを一つ選んでください。
2）各項目の反応がどれくらいの期間続いているのかについて、最も近いものを一つ選んでください。
持続期間は、1ヶ月未満の場合もあれば、1-6ヶ月、または6ヶ月を超えるかもしれません。この見積もりは簡単ではありませんが、反応の持続時間を大まかに分類してみてください。

		ここ1週間の頻度				持続期間		
		全くない	めったにない	たまにある	よくある	1ヶ月未満	1-6ヶ月	6ヶ月超2年未満
1	私はそのストレスフルな問題が生じて以降、気分が落ち込み、悲しい。	1	2	3	4	□	□	□
2	私はそのストレスフルな問題について、繰り返し考えてしまう。	1	2	3	4	□	□	□
3	私はそのストレスフルな問題について、話すことを可能な限り避けるようにしている。	1	2	3	4	□	□	□
4	私はそのストレスフルな問題を考えてばかりで、大きな負担となっている。	1	2	3	4	□	□	□
5	近頃、以前楽しんでいたような活動は、めったにしない。	1	2	3	4	□	□	□
6	私はそのストレスフルな問題を考えると、本当に不安な状態となる。	1	2	3	4	□	□	□
7	私はそのストレスフルな問題を想起させるかもしれない、特定の出来事や状況を避けている。	1	2	3	4	□	□	□
8	私はそのストレスフルな問題が生じて以降、緊張感は強く、落ち着かない。	1	2	3	4	□	□	□
9	私はそのストレスフルな問題が生じて以降、小さなことでもすぐに短気を起こしてしまう。	1	2	3	4	□	□	□
10	私はそのストレスフルな問題が生じて以降、特定のことに集中するのが難しい。	1	2	3	4	□	□	□

		ここ１週間の頻度				持続期間		
		全く ない	めったに ない	たまに ある	よく ある	1ヶ月 未満	1-6ヶ月	6ヶ月超 2年未満
11	私は自分の記憶からそのストレスフルな問題を、消し去ろうとしている。	1	2	3	4	□	□	□
12	私はそのストレスフルな問題のため、イライラしやすくなっていることに気がついている。	1	2	3	4	□	□	□
13	私はそのストレスフルな問題を常に思い出して、それを止めるすべが何もない。	1	2	3	4	□	□	□
14	私は自分の感情が重荷になるので、それを抑圧している。	1	2	3	4	□	□	□
15	私の思考は、そのストレスフルな問題に関係するものを中心に展開されてしまう。	1	2	3	4	□	□	□
16	私はそのストレスフルな問題が生じて以降、特定のことをしたり、特定の状況が怖い。	1	2	3	4	□	□	□
17	私はそのストレスフルな問題が生じて以降、仕事に行くのも、日常生活で必要なことをこなすのも嫌になっている。	1	2	3	4	□	□	□
18	私はそのストレスフルな問題が生じて以降、意気消沈し、将来への希望はほとんどない。	1	2	3	4	□	□	□
19	私はそのストレスフルな問題が生じて以降、まともに眠れなくない。	1	2	3	4	□	□	□
20	私の個人的な人間関係や余暇活動、あるいは人生の重要な側面にそのストレスフルな問題は、強く影響を与えている。	1	2	3	4	□	□	□

ADNM-20
合計得点 □

ADNM-20 採点シート

適応反応症の症状重症度

ここ１週間の頻度に関する全20項目の得点の総和を計算し、ADNM-20総得点を算出する
（最小：20点-最高：80点）
※「持続期間」の項目は、目的に応じて省略可能。

症状カテゴリー（下位尺度）

中核症状：

- ストレス因子へのとらわれ　*2, 4, 13, 15の各項目の合計点*
- 適応の失敗　*10, 17, 19, 20の各項目の合計点*

随伴症状：

- 回避　*3, 7, 11, 14の各項目の合計点*
- 抑うつ気分　*1, 5, 18の各項目の合計点*
- 不安　*6, 16の各項目の合計点*
- 衝動性の問題　*8, 9, 12の各項目の合計点*

Original Validation:

Glaesmer, H., Romppel, M., Braehler, E., Hinz, A., & Maercker, A. (2015). Adjustment Disorder as proposed for ICD-11: Dimensionality and Symptom Differentiation. *Psychiatry Research, 229*, 940 - 948

Further references:

Bachem, R., Perkonigg, A., Stein, D. J., & Maercker, A. (2016). Measuring the ICD-11 adjustment disorder concept: Validity and sensitivity to change of the Adjustment Disorder - New Module questionnaire in a clinical intervention study. *International Journal of Methods in Psychiatric Research*, 1–9.

Einsle, F., Köllner, V., Dannemann, S., & Maercker, A. (2010). Development and validation of a self-report for the assessment of adjustment disorders. Psychology, Health & Medicine, 15(5), 584–95.

Glaesmer, H., Romppel, M., Braehler, E., Hinz, A., & Maercker, A. (2015). Adjustment Disorder as proposed for ICD-11: Dimensionality and Symptom Differentiation. *Psychiatry Research, 229*, 940 – 94.

Lorenz, L. (2016). *Diagnostik von Anpassungsstörungen. Ein Fragebogen zum neuen ICD-11-Modell.* Wiesbaden: Springer Fachmedien.

Lorenz, L., Bachem, R., & Maercker, A. (2015). The Adjustment Disorder – New Module 20 as a Screening Instrument: Cluster Analysis and Cut-Off Values. *International Journal of Occupational and Environmental Medicine, 7,* 215-220.

Lorenz, L., Hyland, P., Maercker, A., & Ben-Ezra, M. An Empirical Assessment of Adjustment Disorder as proposed for ICD-11 in a General Population Sample of Israel. *Journal of Anxiety Disorders, 54,* 65-70.

Maercker, A., Einsle, F., Köllner, V. (2007). Adjustment disorders as stress response syndromes: A new diagnostic concept and its exploration in a medical sample. *Psychopathology*, *40*, 135 – 146.
